# Apocrine-Eccrine Carcinomas: Molecular and Immunohistochemical Analyses

**DOI:** 10.1371/journal.pone.0047290

**Published:** 2012-10-09

**Authors:** Long P. Le, Dora Dias-Santagata, Amanda C. Pawlak, Arjola K. Cosper, Anh Thu Nguyen, M. Angelica Selim, April Deng, Nora K. Horick, A. John Iafrate, Martin C. Mihm, Mai P. Hoang

**Affiliations:** 1 Department of Pathology, Massachusetts General Hospital and Harvard Medical School, Boston, Massachusetts, United States of America; 2 Department of Pathology, Duke University Medical Center, Durham, North Carolina, United States of America; 3 Department of Pathology, University of Massachusetts (UMass) Medical Center, Worcester, Massachusetts, United States of America; 4 Biostatistics Center, Massachusetts General Hospital, Boston, Massachusetts, United States of America; 5 Department of Dermatology, Brigham and Women’s Hospital, Boston, Massachusetts, United States of America; Johns Hopkins University, United States of America

## Abstract

Apocrine-eccrine carcinomas are rare and associated with poor prognosis. Currently there is no uniform treatment guideline. Chemotherapeutic drugs that selectively target cancer-promoting pathways may complement conventional therapeutic approaches. However, studies on genetic alterations and EGFR and Her2 status of apocrine-eccrine carcinomas are few in number. In addition, hormonal studies have not been comprehensive and performed only on certain subsets of apocrine-eccrine carcinomas. To investigate whether apocrine-eccrine carcinomas express hormonal receptors or possess activation of oncogenic pathways that can be targeted by available chemotherapeutic agent we performed immunohistochemistry for AR, PR, ER, EGFR, and HER2 expression; fluorescence *in situ* hybridization (FISH) for *EGFR* and *ERBB2* gene amplification; and molecular analyses for recurrent mutations in 15 cancer genes including *AKT-1*, *EGFR, PIK3CA*, and *TP53* on 54 cases of apocrine-eccrine carcinomas. They include 10 apocrine carcinomas, 7 eccrine carcinomas, 9 aggressive digital papillary adenocarcinomas, 10 hidradenocarcinomas, 11 porocarcinomas, 1 adenoid cystic carcinoma, 4 malignant chondroid syringomas, 1 malignant spiradenoma, and 1 malignant cylindroma. AR, ER, PR, EGFR and HER2 expression was seen in 36% (19/53), 27% (14/51), 16% (8/51), 85% (44/52) and 12% (6/52), respectively. Polysomy or trisomy of EGFR was detected by FISH in 30% (14/46). Mutations of *AKT-1*, *PIK3CA*, and *TP53* were detected in 1, 3, and 7 cases, respectively (11/47, 23%). Additional investigation regarding the potential treatment of rare cases of apocrine-eccrine carcinomas with PI3K/Akt/mTOR pathway inhibitors, currently in clinical testing, may be of clinical interest.

## Introduction

In a recent World Health Organization (WHO) classification of cutaneous appendageal carcinomas; apocrine-eccrine, follicular, and sebaceous carcinomas were the three main categories cited in the consensus classification after taking into account the clinical, histologic, and molecular genetic features [1]. Apocrine-eccrine carcinomas are rare and associated with poor prognosis [2]–[4]. Three of nine cases of clear cell eccrine carcinomas reported by Wong *et al.* [4] developed metastases despite local excision, radiation, and chemotherapy. In the largest series of 69 cases of porocarcinomas by Robson *et al*. [3], 17% and 19% experienced local recurrence and lymph node metastases, respectively. In recent Surveillance, Epidemiology, and End Results (SEER) Program of the National Cancer Institute data from 1978 through 2005, the incidence rate of apocrine-eccrine carcinomas was reported to be 2.6 per 1 million person-years [2]. The five-year relative survival rates for apocrine-eccrine carcinomas were 99% for localized, 94% for regional and 51% for distant disease [2].

Due to the frequent tendency for recurrence (50%) and potential to metastasize (14%), amputation of the digit is often the treatment for aggressive digital papillary adenocarcinoma [5]. For the remaining apocrine-eccrine carcinomas, wide surgical excision is also the treatment of choice. Currently there is no uniform guideline concerning the treatment for various types of apocrine-eccrine carcinomas, especially for those with metastases. Treatment success for metastatic disease has been documented only in isolated case reports of metastasizing hidradenocarcinomas and eccrine carcinoma [6]–[8].

Targeted therapy may be a potential treatment option in patients whose tumors are characterized by a relevant oncogene mutation [4], [9], [10]. In a growing number of tumor types including breast, colorectal and lung cancer, selective agents that target critical cancer-promoting pathways are now the treatment of choice for those patients carrying the genetic changes recognized by the drugs [4], [9], [10]. Members of the ERBB receptor tyrosine kinase family, including epidermal growth factor receptor (EGFR), HER2, HER3 and HER4, also present possible targeted therapeutic options [11]. *ERBB2* (v-erb-b2 erythroblastic leukemia viral oncogene homolog 2) gene amplification and response to trastuzumab were documented in a case of metastasizing hidradenocarcinoma [7]. The membranous expression of these markers has therapeutic implications and second-generation epidermal growth factor receptor tyrosine kinase inhibitors such as HKI-272, XL647, and BIBW2992 have dual activity, inhibiting both EGFR and HER2 receptors [12], [13]. Targeting the hormone receptor pathway may also represent one potential therapeutic approach [14].

With the exception of the tumors associated with familial syndromes, studies on genetic alterations [15]–[19] and biomarkers such as epidermal growth factor receptor (EGFR) and HER2 status [7], [17], [20] of apocrine-eccrine carcinomas are few in number. Translocation t(11;19) has been demonstrated in 2 of 11 hidradenocarcinomas [17]. In addition, hormonal studies have not been comprehensive and performed only on certain subsets of apocrine-eccrine carcinomas [7], [21]–[23].

We have previously reported mutations of *PIK3CA* and *TP53* in 2 and 3 tumors, respectively, in a series of 14 metastasizing apocrine-eccrine carcinomas [16]. Only one case of aggressive digital papillary adenocarcinoma was included in that study. In addition, subtypes such as adenoid cystic carcinoma, malignant chondroid syringoma, malignant spiradenoma, and malignant cylindroma were not included in our previous series [16]. To investigate whether apocrine-eccrine carcinomas express hormonal receptors or possess activation of oncogenic pathways that could be targeted by available chemotherapeutic agent we performed immunohistochemistry for AR, ER, PR, EGFR, and HER2 expression; fluorescence *in situ* hybridization (FISH) for *EGFR* and *ERBB2* gene amplification; and single base extension genotyping [24] for recurrent mutations in 15 cancer genes including *AKT-1*, *EGFR, PIK3CA*, and *TP53* on an expanded series of 54 cases of apocrine-eccrine carcinomas (**Table 1**).

**Table 1:** SNaPshot® mutational assays [24].

Gene	Amino Acid – cDNA residue	Gene	Amino Acid –cDNA residue
**v-akt murine thymoma viral oncogene** **homolog1 (** ***AKT1*** **)**	E17–49G	***NOTCH1***	L1575–4724T
**Adenomatous polyposis coli (** ***APC*** **)**	R1114–3340C		L1601–4802T
	Q1338–4012C	**Neuroblastoma RAS viral (v-ras)** **oncogene homolog (** ***NRAS*** **)**	G12–34G
	R1450–4348C		G12–35G
	T1556fs* - 4666_4667insA		G13–37G
**v-raf murine sarcoma viral oncogene** **homolog B1 (** ***BRAF*** **)**	V600–1798G		G13–38G
	V600–1799T		Q61–181C
**Catenin (cadherin-associated protein),** **beta 1, 88 kDa (** ***CTNNB1*** **)**	D32–94G		Q61–182A
	D32–95A		Q61–183A
	S33–98C	**Phosphoinositide-3-kinase, catalytic,** **alpha polypeptide (** ***PIK3CA*** **)**	R88–263G
	G34–101G		E542–1624G
	S37–109T		E545–1633G
	S37–110C		Q546–1636C
	T41–121A		Q546–1637A
	T41–122C		H1047–3139C
	S45–133T		H1047–3140A
	S45–134C		G1049–3145G
**Epidermal growth factor receptor (** ***EGFR*** **)**	G719–2155G	**Phosphatase and tensin homolog (** ***PTEN*** **)**	R130–388C
	T790–2369C		R173–517C
	L858–2573T		R233–697C
	E746_A750–2235_2249del		K267fs* - 800delA
	E746_A750–2235_2250del	**Tumor protein 53 (** ***TP53*** **)**	R175–524G
**Isocitrate dehydrogenase 1 (NADP+),** **soluble (** ***IDH1*** **)**	R132–394C		G245–733G
	R132–395G		R248–742C
**Fms-related tyrosine kinase 3 (** ***FLT3*** **)**	D835–2503G		R248–743G
**Janus kinase 3 (** ***JAK2*** **)**	V617–1849G		R273–817C
**v-kit Hardy-Zuckerman 4 feline** **sarcoma viral oncogene (** ***KIT*** **)**	D816–2447A		R273–818G
**v-Ki-ras2 Kirsten rat sarcoma viral** **oncogene homolog (** ***KRAS*** **)**	G12–34G		R306–916C
	G12–35G		
	G13–37G		
	G13–38G		

## Materials and Methods

This study has been approved by the Massachusetts General Hospital institutional review board (IRB No. 2011-P-2489). Since the study is limited to the use of excess human material and health related information, written consent was exempted by IRB. Archival materials of all cutaneous apocrine-eccrine carcinomas including apocrine carcinoma, eccrine carcinoma, aggressive digital papillary adenocarcinoma, hidradenocarcinoma, malignant spiradenoma, porocarcinoma, adenoid cystic carcinoma, malignant chondroid syringoma, and malignant cylindroma diagnosed between 1987 and 2011 were retrieved from the pathology files of the Massachusetts General Hospital, Boston, MA. In addition, the personal consultation files of MCM from 2006–2011 was searched. Age, gender, tumor site, tumor size and clinical follow-up information (such as local recurrence or metastasis) were extracted from the patients’ medical records. All patient data were de-identified. The histologic sections of all cases were re-examined and the diagnoses were confirmed.

### Immunohistochemistry

Immunohistochemical studies were performed on five-micrometer-thick sections of formalin-fixed, paraffin-embedded tissue, using the standard techniques involving heat-induced epitope retrieval buffer, and primary antibodies against AR (M3562, 1∶50, Dako, Carpinteria, CA), ER (SP1, prediluted, Ventana Medical Systems, Tucson, AZ), PR (1E2, prediluted, Ventana Medical Systems), EGFR (3C6, prediluted, Ventana Medical Systems), and HER2 (4B5, prediluted, Ventana Medical Systems). Appropriate positive and negative controls were included.

Nuclear expression of AR, ER and PR were graded as percentage of positive cells: 0% = 0, 1–10%  = 1+, 11–25%  = 2+, 26–50%  = 3+, and 51–100%  = 4+. Evaluation of membranous EGFR expression was performed using a combined scoring system based on both the staining intensity (0 = no staining, 1 = weak, 2 = moderate, 3 = strong staining) as well as the percentage of positive cells (0%  = 0, <25%  = 1, 26–50%  = 2, 51–75%  = 3, >75%  = 4), similar to that outlined by Janisson-Dargaud *et al* [25]. The sum of these 2 scores yielded a total score from 0 to 7 (1–3 = weak, 4–7 = strong). Overexpression of HER2 was defined as positive membranous staining in more than 10% of the neoplastic cells. Partial and faint, weak or thin, and intense or thick circumferential membrane staining in more than 10% of the tumor cells were scored as 1+(negative), 2+ (equivocal), and 3+ (positive), respectively.

### Mutational Analysis and *EGFR* and *ERBB2* Fluorescence *in situ* Hybridization (FISH)

A SNaPshot® genotyping assay recently developed by our group was performed on 50 tumors with available archival materials [24]. This assay consists of multiplexed PCR followed by a single-base extension reaction and uses the commercially available SNaPshot platform (Applied Biosystems). The original tumor genotyping panel described by Dias-Santagata *et al* [24], was expanded to include three additional assays (AKT1.49, testing for the *AKT1* E17K mutation; and IDH1.394 and IDH1.395, testing for hotspot mutations in *IDH1*, which affect codon R132). The full panel is outlined in **Table 1** and tests for common mutations in 15 cancer genes. SNaPshot® genotyping was performed using previously described conditions [24], and included the following additional primers for *AKT1* and *IDH1* (PCR: *AKT1* exon 3 Forward, 5′-ACGTTGGATGGGTAGAGTGTGCGTGGCTCT-3′; *AKT1* exon 3 Reverse, 5′- ACGTTGGATGAGGTGCCATCATTCTTGAGG-3′; *IDH1* exon 4 Forward, 5′-ACGTTGGATGGGCTTGTGAGTGGATGGGTA-3′, *IDH1* exon 4 Reverse 5′- ACGTTGGATGGCAAAATCACATTATTGCCAAC-3′. Extension: *AKT1*.49 extR 5′- CTGACTGACTGACTGACTGACTGACTGACTGACTGACTGACTGACTGACTGACTGACTGACTGACTGACTGAGCCAGGTCTTGATGTACT-3′*IDH1*.394 extR 5′-GACTGACTGGACTGACTGACTGACTGACTGGACTGACTGACTGAGATCCCCATAAGCATGAC-3′, *IDH1*.395 extR 5′-TGATCCCCATAAGCATGA-3′).

*EGFR* gene copy number was assessed in 49 tumors by fluorescence *in situ* hybridization (FISH) as previously published [26]. FISH failed in 3 tested cases. Gene amplification and polysomy were defined per criteria outlined by Cappuzzo *et al* [27]. *ERBB2* gene copy number was also assessed in 6 cases with 2+ HER2 protein overexpression. Polysomy 7 was defined as three or more CEP signals per cell.

### Statistical Analysis

For patients with follow-up data available, metastasis-free survival time was calculated as time from diagnosis to identification of metastatic disease in the lymph nodes or distant organs. Patients who did not develop metastases were considered censored at the time of most recent follow-up report. Log-rank tests were used to compare the distribution of metastasis-free survival between subgroups defined by positivity for mutational status (defined as non-wild type for one or more genes tested); *EGFR* FISH; AR, ER, PR, EGFR, and HER2 expression. Kaplan-Meier plots were created to visually assess the differences in metastasis-free survival among subgroups. The statistical association of AR expression in apocrine versus eccrine carcinoma was analyzed by Fisher's Exact Test. A two-tailed *p* value of less than 0.05 was considered to be statistically significant.

## Results

A total of 54 cases were identified: apocrine carcinoma (10), eccrine carcinoma (7), aggressive digital papillary adenocarcinoma (9), hidradenocarcinoma (10), porocarcinoma (11), adenoid cystic carcinoma (1), malignant chondroid syringoma (4), malignant spiradenoma (1), and malignant cylindroma (1).

The age of the patients ranged from 22 to 94 years (median, 62 years). The male to female ratio was 1∶1. The locations of the tumors include: head and neck region (19), axilla (3), finger (9), hand (1), trunk (10), lower extremity (3), foot (3), vulva (4), and lymph node metastases (2). Six patients received radiation therapy (3 with porocarcinomas, 1 aggressive digital papillary adenocarcinoma, 1 eccrine carcinoma and 1 apocrine carcinoma) and one patient with aggressive digital papillary adenocarcinoma received 6 cycles of Carbol/taxol chemotherapy.

Follow-up was available for 38 patients (range, 0–11.4 years; median: 2.6 years). Four cases (one apocrine carcinoma, one eccrine carcinoma, and two porocarcinomas) developed recurrences. Metastases developed in 15 (39%) patients (4 with apocrine carcinoma, 6 eccrine carcinoma, 2 porocarcinomas, 2 aggressive digital papillary adenocarcinomas, and 1 hidradenocarcinoma) (**Table 2**). Widespread metastases developed in one patient with eccrine carcinoma that resulted in death (**Table 2**).

**Table 2:** Clinical data of the fifteen patients with apocrine-eccrine carcinomas and metastatic disease.

	Age/gender	Tumor type	Tumor site	Local recurrence	Lymph node metastasis	Distant metastases	Surgery	Adjuvant therapy
1	45/F	apocrine	L axilla	none	axillary LN	Spine	LN dissection	
2	69/F	apocrine	R vulva	none	5/7 groin LN	None	bilateral LN dissection	
3	81/M	apocrine	axilla	yes	axillary LN	None	LN dissection	radiotherapy
4	38/F	apocrine	vulva	none	L inguinal LN	None	LN dissection	
5	68/F	eccrine	L foot	none	none	R groin skin		
6	62/M	eccrine	L lower leg	none	thoracic LN	lung, liver, adrenal glands		
7	62/M	eccrine	L groin	none	7/9 inguinal LN	Skin	bilateral LN dissection	radiotherapy5-Fluoracil and cisplatinum
8	80/M	eccrine	L cheek	none	3/9 neck LN	None		
9	81/M	eccrine	groin	yes	yes	Skin		
10	66/M	eccrine	L dorsal foot	none	yes	Skin		
11	51/M	ADPA	L index finger	none	L axillary LN	Lung		6 cycles of Taxol/Carbo
12	51/M	ADPA	L index finger	none	yes	None		
13	78/M	hidradenocarcinoma	R shoulder	none	6/22 R axillary LN	None	LN dissection	
14	78/F	porocarcinoma	L lower leg	none	4/6 L inguinal LN	Skin	LN dissection	radiotherapy
15	83/M	porocarcinoma	L ear	yes	yes	None	LN dissection	radiotherapy

F: female, M: male, R: right, L: left, ADPA: aggressive digital papillary adenocarcinoma, LN: lymph node.

### Immunohistochemistry

**Figure 1:**
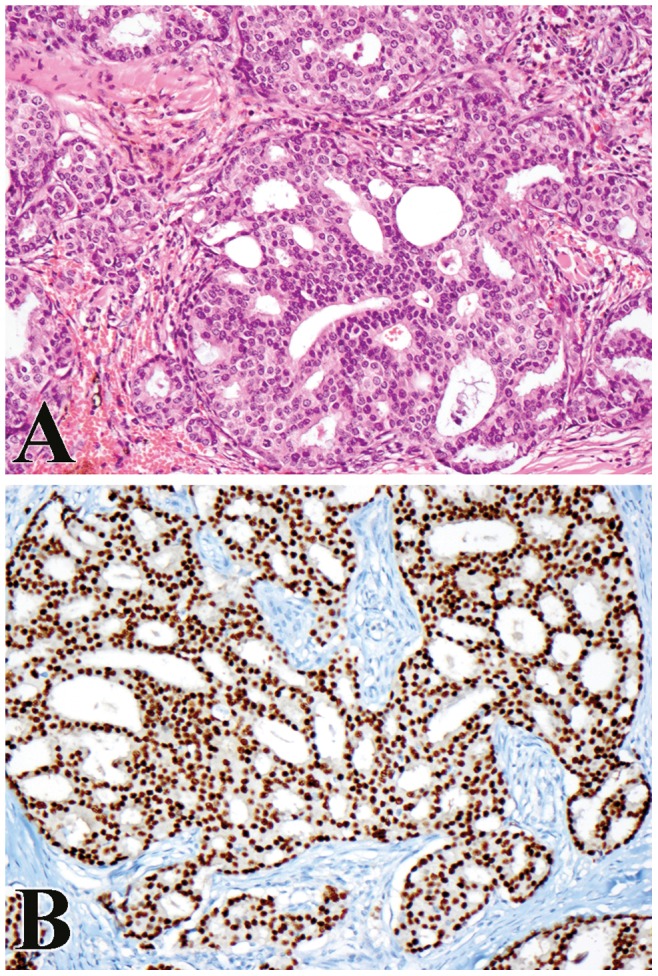
Apocrine carcinoma. (A) Cribriform architecture, polygonal neoplastic cells and eosinophilic cytoplasm are characteristic features of apocrine carcinoma (X200). (B) Strong and diffuse nuclear staining for androgen receptor is noted (X200).

**Figure 2:**
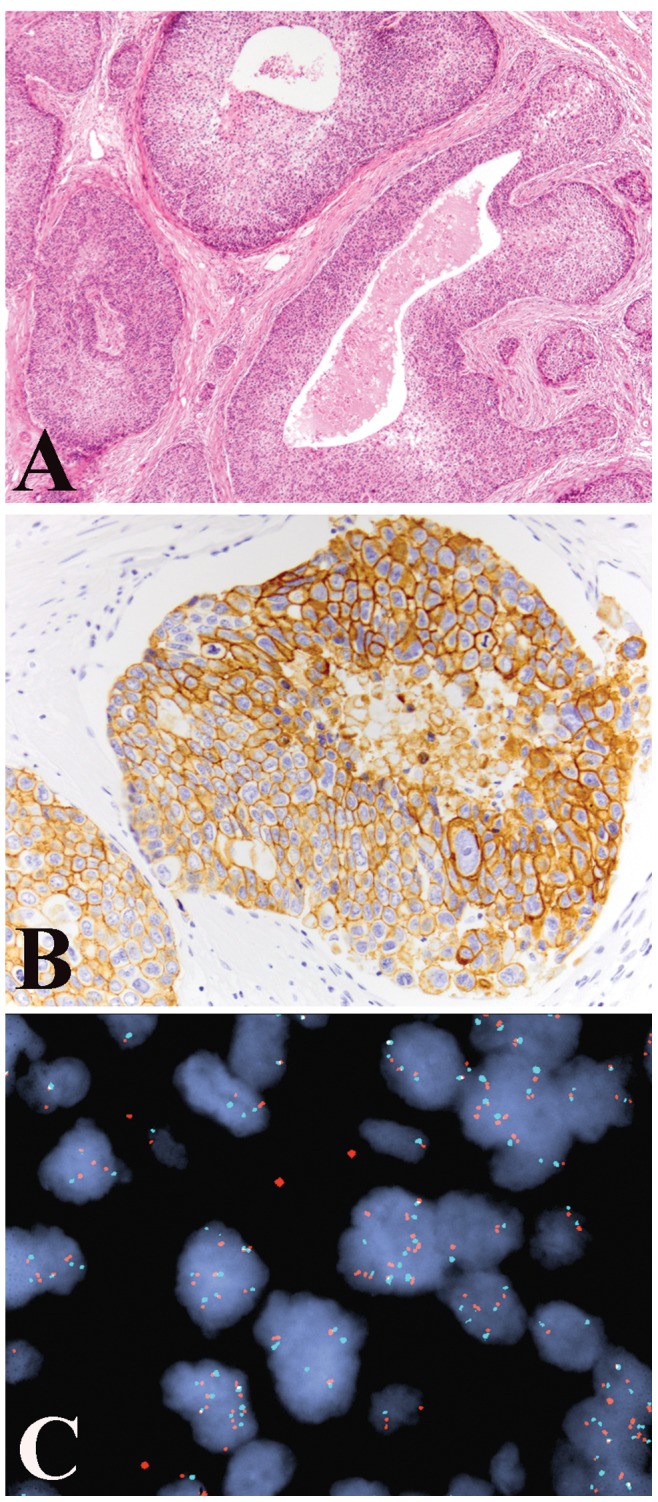
Hidradenocarcinoma. (A) Cribriform necrosis and clear cell change are seen in a hidradenocarcinoma (X40). (B) Strong membranous expression of EGFR was noted (X200). (C) However, fluorescence *in situ* hybridization revealed only balanced polysomy of chromosome 7 and *EGFR* gene (X1000).

Immunohistochemistry was performed on 54 cases and the results are summarized in **Table 3**. Forty three percent (23/53) of cases expressed 1–4+ AR (**Figures 1A and 1B**). Thirty three percent (17/51) of cases expressed 1–4+ ER. Twenty percent (10/51) of apocrine-eccrine carcinomas expressed 1–4+ PR. Significant p-value (p<0.0001 via Fisher’s exact test) was noted when comparing AR-positivity in apocrine carcinoma versus eccrine carcinoma. Overexpression of EGFR was seen in 44/52 (85%) cases (**Figures 2A and 2B**), with 40 cases showing high and 4 cases showing low level of expression. HER overexpression (2+) was seen in 6/52 (12%) cases. Metastasis-free survival did not differ significantly by expression of AR, ER, PR, EGFR, or HER2 (p>0.05 for all comparisons).

**Table 3:** Summary of immunohistochemical analyses in apocrine-eccrine carcinomas.

	N	AR		ER		PR		EGFR		HER2	
		1–4+	0	1–4+	0	1–4+	0	Low/High	0	2+	1+/0
**Apocrine carcinoma**	10	9	0	5	4	2	7	5	5	3	7
**Eccrine carcinoma**	7	0	7	1	6	3	4	6	1	0	7
**Aggressive digital papillary adenocarcinoma**	9	6	3	2	7	1	8	9	0	1	8
**Hidradenocarcinoma**	10	2	8	1	9	1	9	9	1	1	9
**Porocarcinoma**	11	3	8	5	4	0	9	9	0	0	9
**Adenoid cystic carcinoma**	1	0	1	1	0	1	0	1	0	0	1
**Malignant chondroid syringoma**	4	3	1	1	3	1	3	3	1	1	3
**Malignant spiradenoma**	1	0	1	0	1	1	0	1	0	0	1
**Malignant cylindroma**	1	0	1	1	0	0	1	1	0	0	1
**Total**	54	**23/53 (43%)**	30/53 (57%)	**17/51 (33%)**	34/51 (67%)	**10/51 (20%)**	41/51 (80%)	**44/52 (85%)**	8/52 (15%)	**6/52 (12%)**	46/52 (88%)

### Mutational Analysis and FISH

**Figure 3:**
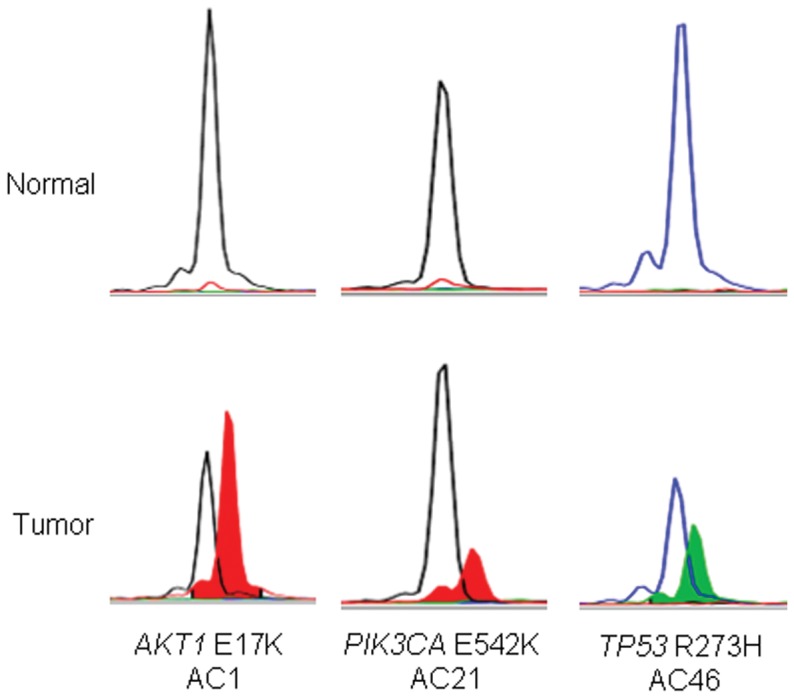
Mutational profiling of apocrine-eccrine carcinoma using SNaPshot® genotyping. The top panel shows genotypic data obtained with normal male genomic DNA (Promega, Madison, WI, USA) and the lower panel illustrates mutation detection in tumor DNA derived from formalin-fixed paraffin-embedded specimens.

**Figure 4:**
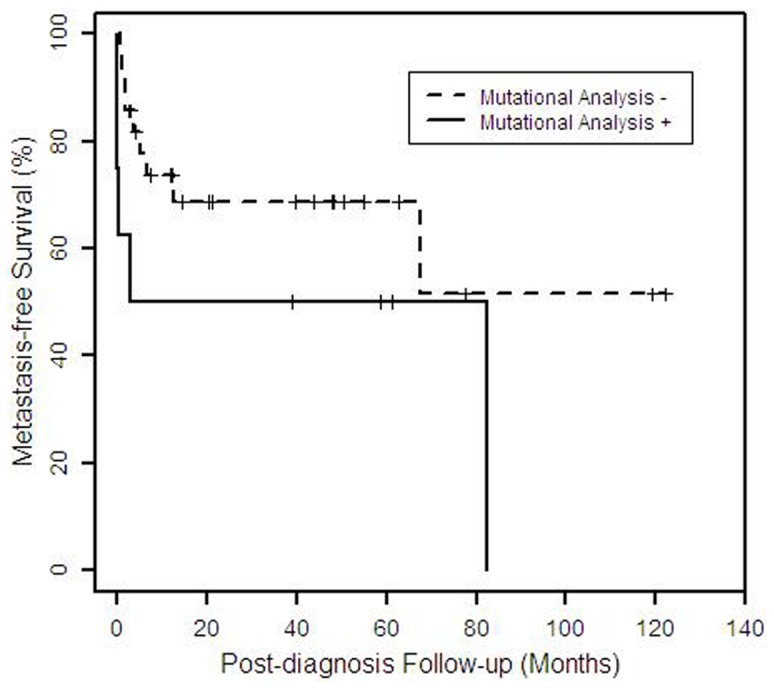
Kaplan-Meier plot of metastasis-free survival time by mutational analysis results (N = 36). The plot shows the distribution of time from diagnosis to metastasis for patients with (n = 8) and without (n = 28) one or more detected mutations, for whom follow-up information is available (N = 36). Patients who did not have metastases are censored (indicated by vertical mark) at the time of the most recent follow-up information.

The molecular results are summarized in **Table 4**. We have recently developed a multiplexed tumor genotyping clinical assay that uses the SNaPshot platform from Applied Biosystems [24]. This assay performs well with archived tissue and tests for recurrent mutations in 15 cancer genes, including potentially actionable targets such as *BRAF*, *EGFR*, *KRAS, PIK3CA*, and *TP53* (**Table 1**). The genes included in this panel were selected based on their clinical significance and on the availability of therapeutic agents (either FDA-approved or under clinical testing) targeting these cancer pathways [24]. Due to poor quality DNA, the assay completely failed in 3 cases. In 5 cases, only a portion of the assay failed (see Appendix S1). SNaPshot genotyping identified somatic mutations in 23% (n = 11) of 47 cases (**Figure 3**). Activating *PIK3CA* mutations were detected in 1 hidradenocarcinoma (c.1624G>A; p.Glu542Lys) and 2 porocarcinomas (c.1624G>A; p.Glu542Lys and c.1633G>A; p.Glu545Lys). *TP53* mutations were detected in 2 eccrine carcinomas (c.743G>A; p.Arg248Gln and c.817C>T; p.Arg273Cys), 2 hidradenocarcinomas (c.817C>T; p.Arg273Cys), 2 aggressive digital papillary adenocarcinoma (c.818G>A; p.Arg273His and c.817C>T; p.Arg273Cys), and 1 malignant cylindroma (c.818G>A; p.Arg273His). *AKT-1* (E17K) mutation was detected in one hidradenocarcinoma. Metastasis-free survival was not significantly associated with mutational status (p = 0.12, **Figure 4**).

**Table 4:** Summary of FISH and mutational analyses in apocrine-eccrine carcinomas.

	*EGFR* FISHpolysomy	*EGFR* FISHtrisomy	*EGFR* FISHpolysomy/trisomy	*TP53* mutation	*PIK3CA*mutation	*AKT-1* mutation
**Apocrine carcinoma**	**1/7**	0/7	**1/7**	0/5	0/5	0/5
**Eccrine carcinoma**	**4/7**	0/7	**4/7**	**2/7**	0/7	0/7
**Aggressive digital papillary adenocarcinoma**	0/8	0/8	0/8	**2/8**	0/8	0/8
**Hidradenocarcinoma**	**2/8**	**2/8**	**4/8**	**2/10**	**1/10**	**1/10**
**Porocarcinoma**	**2/9**	**1/9**	**3/9**	0/11	**2/11**	0/11
**Adenoid cystic carcinoma**	0/1	0/1	0/1	0/1	0/1	0/1
**Malignant chondroid syringoma**	0/4	0/4	0/4	0/3	0/3	0/3
**Malignant spiradenoma**	**1/1**	0/1	**1/1**	0/1	0/1	0/1
**Malignant cylindroma**	0/1	**1/1**	**1/1**	**1/1**	0/1	0/1
**Total**	**10/46 (21%)**	**4/46 (9%)**	**14/46 (30%)**	**7/47 (15%)**	**3/47 (6%)**	**1/47 (2%)**

**Figure 5:**
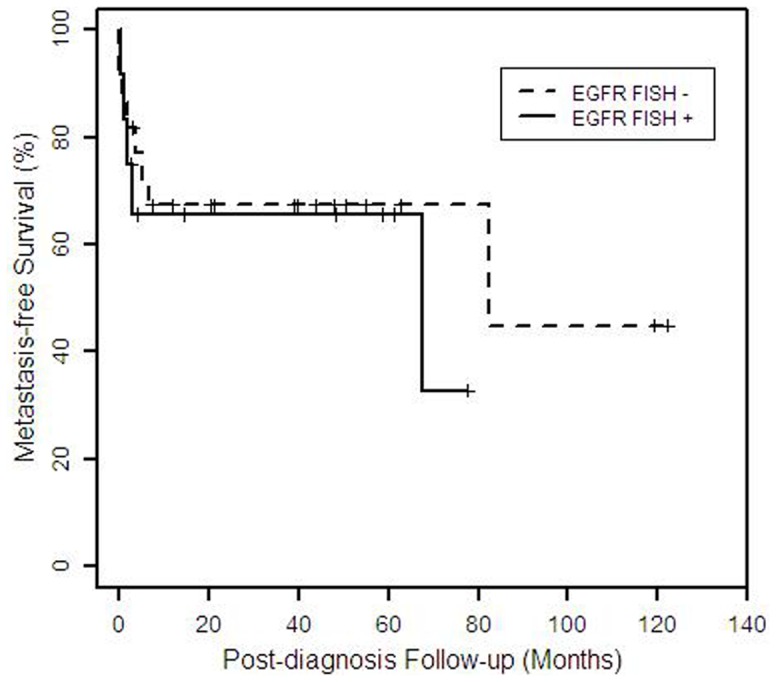
Kaplan-Meier plot of metastasis-free survival time by *EGFR* FISH results (N = 34). The plot shows the distribution of time from diagnosis to metastasis for patients with (n = 12) and without (n = 22) polysomy/trisomy for EGFR, for whom follow-up information is available (N = 34). Patients who did not have metastases are censored (indicated by vertical mark) at the time of the most recent follow-up information.

FISH for *EGFR* gene amplification was successful in 46/50 cases. Either polysomy (**Figure 2C**) or trisomy was detected in 14/46 (30%) cases. Of these 14 cases, 4 (9%) exhibited high polysomy of *EGFR* (defined as ≥4 copies in ≥40% of cells by Capuzzzo *et al* [27]). Although 84% of cases overexpressed EGFR, only 30% exhibited polysomy or trisomy and no cases showed gene amplification. ERBB2 gene amplification was not detected in any of the 6 cases with 2+ HER2 protein expression. Thus, there appears to be no correlation between IHC and FISH in evaluating both EGFR and ERBB2. There was no significant difference in the distribution of metastasis-free survival time comparing patients with detected polysomy/trisomy in EGFR FISH compared to those without (p = 0.6, **Figure 5**).

## Discussion

It is known that apocrine-eccrine carcinomas often express estrogen receptor and progesterone receptor [21], [23]. Due to their similar morphology and possible embryologic origin, it is not surprising that ER, PR, and AR expression can be seen in apocrine-eccrine carcinomas as observed in mammary carcinomas [21]–[23], [28], [29]. ER expression was seen in 10/33 (30%) apocrine-eccrine carcinomas in a series by Swanson *et al* [23] and the positive tumors included 8 eccrine carcinomas, 1 porocarcinoma, and 1 mucinous eccrine carcinoma. Twenty-one percent (9/42) and nineteen percent (8/42) of primary sweat gland carcinomas expressed ER and PR, respectively, in a series by Busam *et al* [21]. Similarly, in our series 33% (17/51) and 20% (10/51) of apocrine-eccrine carcinomas expressed ER and PR, respectively. The expression of hormonal receptors could have treatment implications since hormonal modulation plays an important role in the prevention and treatment of breast carcinomas [14]. Choudhry *et al* [30] reported AR expression in normal apocrine as well as eccrine glands. This suggests that androgen may modulate the function of both apocrine and eccrine glands. The expression of steroid receptors in aggressive digital papillary adenocarcinomas has not been studied. Of interest, all of the primary cutaneous apocrine carcinomas (9/9, 100%) and a third of the aggressive digital papillary adenocarcinomas express AR in more than 10% of tumor cells in our current series. By contrast, none of the eccrine tumors (0/7, 0%) in our series exhibited AR expression. This suggests that AR-positivity is strongly correlated with pure apocrine carcinoma morphology (p<0.0001). This raises the potential of anti-androgen therapy for these subsets of apocrine-eccrine carcinomas. Androgen deprivation therapy using bicalutamide has been reported to be beneficial in the treatment of metastasizing salivary duct carcinoma [31].

Strong (3+) overexpression of HER2 (3+) and gene amplification have been documented in one case of metastasizing hidradenocarcinoma [7]. These findings suggested that *ERBB2* may be a relevant therapeutic target in rare cases of apocrine-eccrine carcinoma; however, we were unable to confirm this finding. None of the six apocrine-eccrine carcinomas in our series with (2+) HER2 overexpression demonstrated *ERBB2* gene amplification. This is consistent with prior published results indicating that high level *ERBB2* gene amplification is unlikely in the setting of 2+ HER2 overexpression [17].

Epidermal growth factor receptor (EGFR/erbB-1) belongs to a receptor family with tyrosine kinase activity whose gene is located on chromosome 7p12. The EGFR signaling that mediates proliferation, migration, invasion, and suppression of apoptosis, can be blocked by a growing number of inhibitor drugs. The role of EGFR inhibitor therapy in apocrine-eccrine carcinomas with protein overexpression remains unclear. Although the majority (85%, 44/52) of apocrine-eccrine carcinomas in our series demonstrated EGFR protein overexpression, only *EGFR* trisomy or polysomy (13/47, 28%) and no gene amplification were documented by FISH, most frequently noted in eccrine carcinoma, hidradenocarcinoma and porocarcinoma. This is not an unexpected finding; since similar results were noted in our recent study of hidradenocarcinomas [20]. In addition, other studies have shown that EGFR overexpression appears to be independent of *EGFR* mutation [32], [33]. In lung and salivary gland carcinomas, high polysomy of *EGFR* if considered to be FISH-positive and the patients would receive treatment [34], [35]. Further studies are warranted to determine whether similar practice may be applied for apocrine-eccrine carcinomas.

Tumor suppressor gene, *TP53*, located on the short arm of chromosome 17p13 has been implicated in the regulation of cell growth, DNA repair, and apoptosis. The *TP53* gene is frequently (14–52%) altered in human breast carcinomas and is the most commonly mutated gene in human tumors [36]–[38]. *TP53* mutations are usually clustered within the most conserved regions of exons 4, 5, 7, and 8 [39]. *TP53* mutations have been previously described in apocrine-eccrine carcinomas (**Table 5**) [15]–[19]. Takata *et al* [18] performed PCR based assays for loss of heterozygosity on chromosomes 3p, 5q, 9p, 9q, 13q, and 17p; yet found only 1 case with *TP53* mutation by direct sequencing of exons 5 to 8. Biernat *et al* [15] employed single-stranded conformation polymorphism analysis followed by direct DNA sequencing and found 5 of 16 sweat gland carcinomas possessed *TP53* mutations (**Table 5**). Two of eleven hidradenocarcinomas exhibited *TP53* mutations in the series by Kazakoz *et al*. [17] Similar to findings reported by Biernat *et al* [15] and Kazakov *et al* [17], we found *TP53* mutations in codon 248 of exon 7 and codon 273 of exon 8 in our 7 positive cases (**Table 5**). Interestingly, these same mutations have also been reported in breast carcinomas [40]–[42]. Although SnaPshot testing works well for oncogenes, which are typically mutated at only a few distinct loci, it is not comprehensive for tumor suppressors. It captures only a few of the numerous mutation events described for TP53, at loci that are not covered by the assay.

**Table 5:** Summary of *TP53* mutations in apocrine-eccrine carcinomas [15]–[19].

Case	Histologic type	*TP53* mutation	Reference
1	aggressive digital papillary adenocarcinoma	Exon 8, Arg273His	16
2		Exon 8, Arg273Cys	current study
3	eccrine carcinoma	Exon 5, Cys176Arg	18
4		Exon 7, Arg248Gln	16
5		Exon 8, Arg273Cys	16
6	hidradenocarcinoma	Exon 5, Cys176Tyr	15
7		Exon 7, Arg248Gln	15
8		Exon 8, Arg273His	17
9		Exon 6, Arg196X & Arg213X	17
10		Exon 8, Arg273Cys	current study
11		Exon 8, Arg273Cys	current study
12	malignant cylindroma	Exon 8, Arg273His	current study
13	Porocarcinoma	Exon 8, codons 273–275, 9bp deletion	15
14	spiradenocarcinoma	Exon 8, Glu285Lys	15
15		Exon 7, Arg248Gln	15
16	trichilemmal carcinoma	Exon 8, codon 306, C → T	19

*PIK3CA* (phosphatidylinositol 3-kinase, catalytic, alpha polypeptide) mutations in these tumors have been reported by our group [16]. In breast carcinomas, the majority of mutations have been identified in the helical domain (exon 9, 37%) and in the kinase domain (exon 20, 63%) of *PIK3CA* [43]. All mutations were single-based substitutions [43]. Similarly we detected 2 types of mutations, c.1624G>A:p.Glu542Lys (in one hidradenocarcinoma and one porocarcinoma) and c.1633G>A:Glu545Lys (in one porocarcinoma), in exon 9 of *PIK3CA*. These 2 mutations are among the three most frequently reported mutations in breast cancer [43]. Mutations in codon 545 represent a mutational hotspot reported in ovarian and colorectal carcinomas as well [44], [45]. The clustering of mutations within *PIK3CA* may prove useful for therapeutic purposes. The phosphatidylinositol 3 kinase (PIK3) signaling pathway is an important regulator of cell growth, proliferation, cell motility, angiogenesis, and survival, and it has been shown that *PIK3CA* is the most frequently mutated gene in breast cancer [46], [47]. It is thought that in breast cancer, oncogenic mutations in *PIK3CA* or low levels of *PTEN* expression may confer resistance to treatment with trastuzumab, a monoclonal antibody that targets the HER2/Neu receptor [48]. *ERBB2* amplification and *PIK3CA* mutation were validated as biomarkers for sensitivity to the single-agent phosphoinositide 3-kinase (PIK3) inhibitor, GDC-0941, in breast cancer models [49]. Other studies have shown that cancers with *PIK3CA* mutations were sensitive to single-agent *PI3K* inhibitors and dual *PI3K*-mammalian target of rapamycin (mTOR) inhibitors [50], [51].

*AKT-1* mutation has not been previously described in cutaneous appendageal carcinomas. In carcinomas, the PI3K/AKT pathway is well characterized [46], [52]; and the PI3K/Akt/mTOR pathway has been shown to be a target for cancer therapy including breast carcinoma [53]. While mutations in the *PI3* kinase gene (*PIK3CA*) are common in breast carcinoma, somatic mutations in *AKT*s are rare [54], [55]. Similarly we found only 1 of 47 cases exhibiting *AKT-1* mutation.

The presence of either high polysomy, low polysomy or trisomy of *EGFR* does not appear to correlate with metastatic disease (p = 0.6, **Figure 5**). Five of the 8 cases (63%) with detected mutations had metastatic disease, while 9 of the 28 cases (32%) with no mutations detected developed metastases; however, there was no significant difference in metastasis-free survival (p = 0.12, **Figure 4**). Thus, there appears to be no correlation between the presence of mutations and metastatic disease. Apocrine-eccrine carcinomas and breast carcinomas are analogous tumors often with similar histology. It is interesting that in this study we detected mutations in *PIK3CA* and *TP53* in a subset of apocrine-eccrine carcinomas, a therapeutically-relevant finding since both of these genes are known to be frequently mutated in breast carcinomas.

Currently there is no uniform guideline concerning the treatment for metastatic apocrine-eccrine carcinomas and treatment success has been documented only in isolated case reports [6]–[8]. A variety of chemotherapeutic agents have been used with varying degrees of responsiveness. Combinations of cyclophosphamide, bleomycin, cisplatin, and 5-fluorouracil; interferon-alpha, interleukin-2, sunitinib, and tamoxifen have been reported with some degree of response [6], [8], [56], [57]. Remission was achieved for 16 months with paclitaxel and carboplatin in a case of apocrine carcinoma with lymph node, lung, and bone metastasis [58]. Hikada et al [59] reported a case of metastatic apocrine carcinoma responded to treatment with HER-2 inhibitors. Radiation therapy has been used in selected cases of metastatic porocarcinoma [60].

In this study we used a SNaPshot platform, previously reported by our group [24], to screen relevant cancer genes with available targeted therapeutic agents in rare tumors. The platform is cost effective due to two main reasons. First, SNaPshot testing uses thermocyclers (PCR machines) and capillary electrophoresis DNA sequencers, which are instruments that already exist in any typical clinical molecular laboratory. Thus, there is not need for an upfront investment in expensive equipment or bioinformatics personnel. Secondly, because SNaPshot tests for hotspot mutations in a multiplexed fashion, it only uses a fraction of tumor tissue required for probing the same number of exons using Sanger sequencing.

In summary, we report mutations in *AKT-1*, *PIK3CA* and *TP53* in a subset of apocrine-eccrine carcinomas including eccrine carcinoma, aggressive digital papillary adenocarcinoma, hidradenocarcinoma, and porocarcinoma. There is strong correlation of AR expression in the apocrine carcinoma subtype, raising the potential for anti-androgen therapy. The role of EGFR inhibitor therapy in apocrine-eccrine carcinomas with protein overexpression remains unclear. The lack of correlation between the protein expression and polysomy/gene amplification suggests that molecular mechanisms other than gene amplification may play a role in EGFR overexpression in adnexal carcinomas. Based on our findings, targeted therapy including PI3K/Akt/mTOR pathway inhibitors, which is currently in clinical testing, may be potential treatment options for rare cases of apocrine-eccrine carcinomas. It will be interesting to determine whether these results will translate into real therapeutic response in followup clinical trial studies.

## Supporting Information

Appendix S1 **Appendix of failed cases.**
(DOC)

Table S1 **Detailed immunohistochemical scorings in apocrine-eccrine carcinomas.**
(DOC)
