# Supplementary material for: Apocrine-Eccrine Carcinomas: Molecular and Immunohistochemical Analyses
Source: PLoS One. 2012 Oct 9;7(10):e47290. doi: 10.1371/journal.pone.0047290 (PMC3467209; doi:10.1371/journal.pone.0047290)
Supplement: Appendix S1 — Appendix of failed cases. (DOC) [file pone.0047290.s001.doc]

**SUPPLEMENTARY MATERIAL**

**Appendix of failed cases**

Hidradenocarcinoma: *KRAS* (34, 35, 37, 38, 181, 182, 183); *NRAS* (34, 35, 37, 38, 181, 182); *PIK3CA* (1633, 3145); *BRAF* (1397, 1406, 1789, 1799); *MAP2K* (167, 171, 199); *TP53* (817); *PTEN* (388)

Hidradenocarcinoma: *KRAS* (34, 181, 183); *NRAS* (37, 181, 182); *NOTCH1* (4724)

Porocarcinoma: *KRAS* (181, 182, 183); *NRAS* (181, 182); *BRAF* (1397, 1406); *MAP2K* (167, 199)

Apocrine carcinoma: *KRAS* (181, 182, 183); *NRAS* (182); *BRAF* (1397, 1406); *MAP2K* (167)

Aggressive digital papillary adenocarcinoma: *KRAS* (181, 183)
