# Supplementary material for: Apocrine-Eccrine Carcinomas: Molecular and Immunohistochemical Analyses
Source: PLoS One. 2012 Oct 9;7(10):e47290. doi: 10.1371/journal.pone.0047290 (PMC3467209; doi:10.1371/journal.pone.0047290)
Supplement: Table S1 — Detailed immunohistochemical scorings in apocrine-eccrine carcinomas. (DOC) [file pone.0047290.s002.doc]

**Table S1: Immunohistochemical scorings in apocrine-eccrine carcinomas**

|  | **N** | **AR** | | | | | **ER** | | | | | **PR** | | | | | **EGFR** | | | **HER2** | |
| --- | --- | --- | --- | --- | --- | --- | --- | --- | --- | --- | --- | --- | --- | --- | --- | --- | --- | --- | --- | --- | --- |
|  |  | **4+** | **3+** | **2+** | **1+** | **0** | **4+** | **3+** | **2+** | **1+** | **0** | **4+** | **3+** | **2+** | **1+** | **0** | **high** | **low** | **0** | **2+** | **1+/0** |
| **Apocrine carcinoma** | 10 | 4 | 4 | 1 | 0 | 0 | 3 | 1 | 1 | 0 | 4 | 1 | 0 | 1 | 0 | 7 | 4 | 1 | 5 | 3 | 7 |
| **Eccrine carcinoma** | 7 | 0 | 0 | 0 | 0 | 7 | 0 | 1 | 0 | 0 | 6 | 0 | 0 | 3 | 0 | 4 | 6 | 0 | 1 | 0 | 7 |
| **Aggressive digital papillary adenocarcinoma** | 9 | 0 | 3 | 0 | 3 | 3 | 0 | 0 | 1 | 1 | 7 | 0 | 1 | 0 | 0 | 8 | 9 | 0 | 0 | 1 | 8 |
| **Hidradenocarcinoma** | 10 | 0 | 1 | 1 | 0 | 8 | 0 | 0 | 1 | 0 | 9 | 0 | 0 | 0 | 1 | 9 | 7 | 2 | 1 | 1 | 9 |
| **Porocarcinoma** | 11 | 0 | 0 | 2 | 1 | 8 | 0 | 3 | 2 | 0 | 4 | 0 | 0 | 0 | 0 | 9 | 9 | 0 | 0 | 0 | 9 |
| **Adenoid cystic carcinoma** | 1 | 0 | 0 | 0 | 0 | 1 | 0 | 0 | 0 | 1 | 0 | 0 | 0 | 0 | 1 | 0 | 1 | 0 | 0 | 0 | 1 |
| **Malignant chondroid syringoma** | 4 | 2 | 1 | 0 | 0 | 1 | 1 | 0 | 0 | 0 | 3 | 0 | 1 | 0 | 0 | 3 | 3 | 0 | 1 | 1 | 3 |
| **Malignant spiradenoma** | 1 | 0 | 0 | 0 | 0 | 1 | 0 | 0 | 0 | 0 | 1 | 0 | 1 | 0 | 0 | 0 | 1 | 0 | 0 | 0 | 1 |
| **Malignant cylindroma** | 1 | 6 | 0 | 0 | 0 | 1 | 0 | 0 | 0 | 1 | 0 | 0 | 0 | 0 | 0 | 1 | 0 | 1 | 0 | 0 | 1 |
